# St. John's Hospital for Diseases of the Skin

**Published:** 1889-06-01

**Authors:** 


### St. John's Hospital for Diseases of the Skin.

The annual general meeting of the governors of this institution was held last week at the Criterion, about 70 people being present. Considerable interest attached to the proceedings in consequence of the undesirable notoriety the hospital has recently attained, owing to the attacks made in the Press against the secretary, and the actions-at-law which followed.

At half-past three a decided struggle commenced, and it was evident enough that two factions were present. Colonel Mercier was proposed to the chair, but an amendment was immediately tabled in favour of the Earl of Aberdeen. His lordship, though denying any feeling of prejudice, was evidently inclined to side with a compaot and vigorous band of medical and other gentlemen who sat together, and were referred to several times as "the opposition."

Colonel Mercier expressed the hope that as the charges against the secretary had resulted in a verdict in his favour, nothing further would be heard from those who were at one time pressing for an enquiry, although, when it was proposed that there should be a committee of enquiry, they had postponed it. It was proposed to recommend that the hospital be registered under the Friendly Societies Act, so that the accounts would be analysed by the Registrar-General, and so could not be discredited.

The Secretary having read the report, Alderman Gould moved its adoption, and referred with much pathos and persistence to the terrible trials and persecutions to which "this poor young man" had been subjected. For the information of the reader it may be added that the poor young man alluded to was Mr. St. Vincent Mercier, a gentleman of six feet odd inches in his socks, and a pair of shoulders that could have easily borne off the worthy alderman, had their owner been so disposed. The libel actions against *Ally Sloper* and *Truth* formed the principal part of Alderman Gould's address, and this speaker took occasion to reprove the Lord Chief Justice of England for his recent behaviour. It was something of a study to listen to this civic dignitary of a remote borough pouring forth words of advice and precept to his Lordship of England. There was a dash of modesty, too, in his manner that made Alderman Gould's strictures more piquant still. "My own decisions," he admitted, with a gracious wave of the hand, "have themselves often been wrong"; and a "hear, hear," from the opposition quarter added an undesired point to the remark. He then complained that Lord Coleridge had allowed an almost forgotten conversation to influence his decision in the action against *Truth*, and maintained that his lordship ought not to have tried the case at all. Mr. T. Savage seconded the adoption of the report, considerable disorder and many interruptions ensuing, the speaker finally concluding abruptly, the impatience of the audience being ill-restrained.

The Earl of Aberdeen then asked (1) why the Hospital Sunday Fund had withheld its grant to the hospital; (2) why the Duke of Northumberland had resigned the presidency? To these questions Colonel Mercier replied (1) that the grant was withheld pending the result of the lawsuits, and would now probably be handed over; (2) though the Duke of Northumberland had resigned, the Duchess had doubled her subscription this year, and the Duke of Newcastle had accepted the presidency.

Dr. Lennox Browne spoke, saying it was not the intention of his friends to propose any hostile resolutions.

Sir Sidney Waterlow stated that the Distribution Committee of the Hospital Sunday Fund were about considering the question of the award to the St. John's and other hospitals. He did not wholly approve of the proposal to register the hospital under the Friendly Societies Act, but did not oppose the adoption of the report. He pointed out that, according to the balance-sheet, the charges for management had come to £845, while the total for maintenance was but £2,007, of which the patients themselves had contributed £1,100. He did not think that these figures would increase the public estimation of this institution as a "charity."

The report was adopted, at length, though not unanimously. The proceedings were stormy throughout.

We can only repeat, what we have said before, that, in our opinion, the public are not justified in subscribing to this hospital unless and until a full enquiry has been held regarding its past transactions. Nothing but such a course can effectually remove the doubts which have arisen in people's minds concerning its management.

